# Plasmid pPCP1-derived sRNA HmsA promotes biofilm formation of *Yersinia pestis*

**DOI:** 10.1186/s12866-016-0793-5

**Published:** 2016-08-04

**Authors:** Zizhong Liu, Xiaofang Gao, Hongduo Wang, Haihong Fang, Yanfeng Yan, Lei Liu, Rong Chen, Dongsheng Zhou, Ruifu Yang, Yanping Han

**Affiliations:** 1 State Key Laboratory of Pathogen and Biosecurity, Beijing Institute of Microbiology and Epidemiology, No. 20, Dongdajie, Fengtai, Beijing, 100071 China; 2 State Key Lab of Space Medicine Fundamentals and Application, China Astronaut Research and Training Center, Beijing, 100094 China; 3 Anhui Medical University, Hefei, Anhui 230032 China; 4 College of Life Sciences, Anhui University, Hefei, Anhui 230601 China; 5 The General Hospital of PLA, Beijing, 100853 China

## Abstract

**Background:**

The ability of *Yersinia pestis* to form a biofilm is an important characteristic in flea transmission of this pathogen. *Y. pestis* laterally acquired two plasmids (pPCP1and pMT1) and the ability to form biofilms when it evolved from *Yersinia pseudotuberculosis.* Small regulatory RNAs (sRNAs) are thought to play a crucial role in the processes of biofilm formation and pathogenesis.

**Results:**

A pPCP1-derived sRNA HmsA (also known as sR084) was found to contribute to the enhanced biofilm formation phenotype of *Y. pestis*. The concentration of c-di-GMP was significantly reduced upon deletion of the *hmsA* gene in *Y. pestis*. The abundance of mRNA transcripts determining exopolysaccharide production, crucial for biofilm formation, was measured by primer extension, RT-PCR and *lacZ* transcriptional fusion assays in the wild-type and *hmsA* mutant strains. HmsA positively regulated biofilm synthesis-associated genes (*hmsHFRS*, *hmsT* and *hmsCDE*), but had no regulatory effect on the biofilm degradation-associated gene *hmsP.* Interestingly, the recently identified biofilm activator sRNA, HmsB, was rapidly degraded in the *hmsA* deletion mutant. Two genes (*rovM* and *rovA*) functioning as biofilm regulators were also found to be regulated by HmsA, whose regulatory effects were consistent with the HmsA-mediated biofilm phenotype.

**Conclusion:**

HmsA potentially functions as an activator of biofilm formation in *Y. pestis*, implying that sRNAs encoded on the laterally acquired plasmids might be involved in the chromosome-based regulatory networks implicated in *Y. pestis*-specific physiological processes.

**Electronic supplementary material:**

The online version of this article (doi:10.1186/s12866-016-0793-5) contains supplementary material, which is available to authorized users.

## Background

The genus *Yersinia* is composed of 11 species, including three human pathogenic species: *Yersinia pestis*, *Yersinia pseudotuberculosis* and *Yersinia enterocolitica. Y. pestis* is thought to have evolved from *Y. pseudotuberculosis* 5021–7022 years ago [1]. Despite >90 % genome identity between *Y. pestis* and *Y. pseudotuberculosis*, the disease caused by these two species differs dramatically. *Y. pseudotuberculosis* is a self-limiting gastroenteric pathogen that does not usually form biofilms [2, 3]. By contrast, *Y. pestis* is a deadly pathogen responsible for three human plague pandemics. It is transmitted to mammals and/or humans by infected flea bites or by direct contact with infected animals [4]. *Y. pestis* must survive and adapt to the complex microenvironments of multiple hosts during its infectious process [4, 5]. During its evolution from *Y. pseudotuberculosis*, *Y. pestis* acquired two unique plasmids, pPCP1 and pMT1, which are crucial for the processes of pathogenesis and flea transmission [6–8]. Plasmid pPCP1 is a 9.5 kb plasmid that encodes the plasminogen activator Pla, a surface protease that is essential for mediating primary pneumonic plague [7, 9].

The formation of biofilm within the flea digestive tract is important for natural transmission of *Y. pestis* because complete blockage of the proventriculus promotes frequent biting by fleas and thus increases the opportunities for transmission [4, 10]. A dense bacterial aggregate embedded in a self-produced exopolysaccharide (EPS) matrix facilitates the adaptation to complex microenvironments [3, 10, 11]. The *hmsHFRS* locus encodes the structural proteins required for the synthesis and transport of EPS, a major component of the *Y. pestis* biofilm [12, 13]. EPS expression is controlled at the post-transcriptional level by the intracellular concentration of the c-di-GMP second messenger [14], which is synthesized by diguanylate cyclases HmsD/HmsT and degraded by the phosphodiesterase HmsP in *Y. pestis* [15–17]. Several transcriptional regulators have been discovered that are involved in biofilm formation in *Y. pestis*. For example, Fur, a regulator of iron metabolism, can repress biofilm formation by negatively regulating the *hmsT* gene [18]. RcsA, a negative regulator of biofilms, is reported to be functionally defective in *Y. pestis* [19]. RovM, which is directly induced under specific microenvironments and represses the expression of the *rovA* gene, also regulates biofilm formation [20]. We recently reported the role of RovA in biofilm formation of *Y. pestis* [21]. The PhoPQ two-component system, a LysR-type transcriptional regulator YfbA and the carbon storage regulator CsrA have recently been shown to contribute to biofilm formation of *Y. pestis* [22–24].

*Y. pestis* has to adapt to diverse environmental conditions during its complex life cycle by modulating the expression of metabolic, cell surface and virulence factors. In bacteria there are different levels at which gene expression can be regulated. Small regulatory RNAs (sRNAs) play important regulatory roles at the post-transcriptional level in bacterial physiology and pathogenesis, including biofilm formation [25]. They are reported to exert their regulatory functions by interacting with specific mRNAs or proteins and thus influence translation and mRNA stability upon sensing environmental cues [26, 27]. The identification of more than 100 sRNAs, identified by RNomics and deep sequencing, facilitates the study of post-transcriptional mechanisms of gene regulation in *Y. pestis* [28–32]. Post-transcriptional regulation and the underlying role of certain novel sRNAs in virulence and host adaptation have begun to be addressed in the genus *Yersinia* in recent years [32–34].

HmsB, a chromosome-encoded sRNA (also known as sR035), was identified by our previous study [28] and subsequently shown to promote biofilm formation by increasing EPS production in *Y. pestis* [35]. The plasmid pPCP1-deriving sRNA HmsA (also known as sR084) was initially found to be highly abundant in *Y. pestis* grown in vitro and positively regulated by the CRP protein, a global regulator of catabolite repression [28]. Here we observed an altered biofilm phenotype in the *hmsA* mutant of *Y. pestis*. Based on results measuring the abundance of mRNA transcripts determining EPS production, HmsA was found to potentially function as an activator of biofilm formation in *Y. pestis* by modulating the intracellular level of c-di-GMP molecules. Interestingly, the recently identified biofilm-associated sRNA, HmsB, was significantly downregulated in the *hmsA* deletion mutant. Furthermore, transcription of the biofilm regulators, RovA and RovM, also seemed to be affected by HmsA, which might partially account for the biofilm phenotype.

## Methods

### Bacterial strains

Bacterial strains, plasmids and primers used in this study are listed in Table 1. *Y. pestis* wild-type (WT) strain 201 belongs to biovar Microtus, which is avirulent in humans but highly virulent in mice [36]. Except for the pPCP1-cured strain, all mutant strains used here were constructed by replacing the entire gene with the kanamycin resistance cassette using the λ-Red homologous recombination system [37]. The pPCP1-cured strain derived from strain 201 was constructed based on plasmid incompatibility by Bin et al. in our laboratory [38]. To obtain the HmsA overexpression strain, the arabinose-inducible transcriptional plasmid pBAD-TF was modified using the QuikChange® Lightning Site-Directed Mutagenesis Kit (Stratagene) as previously described [39]. The HmsA overexpression plasmid (pBAD-HmsA) was constructed by ligating the PCR-generated DNA fragment spanning the full-length *hmsA* gene (65 nt) and the 27-nt downstream region of *hmsA* into the modified vector. The HmsA overexpression strain ∆*hmsA*::HmsA was generated by introducing plasmid pBAD-HmsA into the *hmsA* deletion mutant (∆*hmsA*).

**Table 1:** Bacterial strains, plasmids and oligonucleotides used in this study

Strains		
Name	Description	Sources
WT	*Y. pestis* wild-type strain 201	[36]
WT::pBAD	*Y. pestis* WT strain carrying plasmid pBAD-TF	[39]
∆*pPCP1*	*Y. pestis* WT strain lacking plasmid pPCP1	[38]
∆*hmsA*	*hmsA* deletion mutant derived from *Y. pestis* WT strain 201	[28]
∆*hmsA*::pBAD	∆*hmsA* strain carrying plasmid pBAD-TF	This study
∆*hmsA*::HmsA	∆*hmsA* strain carrying plasmid pBAD-HmsA	This study
*∆hmsS*	*hmsS* deletion mutant derived from *Y. pestis* WT strain	[18]
*∆fur*	*fur* deletion mutant derived from *Y. pestis* WT strain	[18]
*∆hmsB*	*hmsB* deletion mutant derived from *Y. pestis* WT strain	[35]
Plasmids		
pRW50	A low-copy *lacZ* fusion vector	[42]
pBAD-TF	Transcriptional fusion vector modified from pBAD/HisA	[39]
pBAD-HmsA	HmsA overexpressing plasmid by inserting a DNA fragment amplified by primer HmsA-pBAD-F/R into pBAD-TF	This study
Oligonucleotides		
Name	Sequence (5′-3′)	
HmsA-pBAD-F/R	CACGAATTCGCAAAAGTCAGGACTAGACATATTAAC/TTGAAGCTTACCACATAAAAAAGGCCCTCACAGG	
HmsA-NB-F/R	CACGAATTCGCAAAAGTCAGGAC/AATTGTAATACGACTCACTATAGGGCGTTGAAGCTTACCACATAAAAAAGGC	
HmsB-NB-F/R	TGTAATAATAGTCATATCATCGTAAATTTGAATTGTAATACGACTCACTATAGGGCGTGCTTCAGTGGCTCATGTC	
HmsA-PE-R	TCTGTGAGGGCGTATGATAAGG	

### Biofilm formation assays

Three methods were used to evaluate biofilm formation by *Y. pestis*, as described previously [18]. For the rugose colony morphology assay, 5 μL of bacterial glycerol stocks were spotted onto LB plates, which were dried for 2 d at 37 °C and cooled for at least 2 h at room temperature, these plates were incubated at 26 °C for 1 week. The surface morphology of each bacterial colony was photographed.

A crystal violet staining assay was used to detect biofilm formation in vitro. One hundred microliters of bacterial glycerol stock were added to 18 mL of LB medium for cultivation to stationary phase at 26 °C. The bacterial cultures were diluted 1:20 into 18 mL of LB medium for cultivation to an optical density (OD_620_) of about 1.0 and stored at 4 °C for 8 h. The bacterial cultures were diluted 1:20 and 1 mL of the diluted cultures was transferred into the 24-well tissue culture plates; these plates were incubated with shaking at 230 rpm for 24 h at 26 °C. The OD_620_ values of the cultures containing planktonic cells were determined and used for normalization. The pellicle was gently washed three times with H_2_O, and incubated at 80 °C for 15 min. The biofilms were stained with 2 mL of 0.1 % crystal violet for 15 min. The wells were washed three times with 2 mL of H_2_O, the biofilms were dissolved with 2 mL of dimethylsulfoxide for 1 h. Crystal violet staining was determined by the OD_570_ values and the relative amount of biofilm formation was indicated by the OD_570_/OD_620_ values. Three biological replicates and two technical replicates were performed for each strain.

For *Caenorhabditis elegans* killing assays, bacterial biofilm formation in vivo was determined by the percentage of fourth-stage larvae and adults (L4/adult) of *C. elegans* after incubation of nematode eggs on *Y. pestis* lawns. The nematode eggs were collected from lysates of adult *C. elegans* grown on *Escherichia coli* OP50 lawns on NGM agar plates. About 200 to 300 nematode eggs were placed on each bacterial lawn, followed by incubation at 20 °C for 72 h, the number of nematodes, fourth-stage larvae and adults of *C. elegans* were counted. Three biological replicates were performed for each strain.

### Measurement of the intracellular c-di-GMP levels

The intracellular c-di-GMP levels were determined by a chromatography-coupled tandem mass spectrometry (HPLC-MS/MS) method. One hundred microliters of bacterial glycerol stocks were added to 18 mL of LB medium for cultivation to stationary phase at 26 °C. The bacterial cultures were diluted 1:20 into 18 mL of LB medium for cultivation to an OD_620_ of about 1.0, and were then stored at 4 °C for 8 h. The bacterial cultures were diluted 1:20 into 18 mL LB medium for cultivation to middle exponential phase (OD_620_ ≈ 1.0) at 26 °C. Bacterial cultures were harvested for the extraction of c-di-GMP. The samples were loaded onto an API 4000-QTRAP mass spectrometer equipped with an electrospray ionization source (Applied Biosystems). One microliter of bacterial culture was harvested and the protein concentration was determined using a Micro BCA Protein Assay Kit (Thermo Scientific). The c-di-GMP levels were expressed as pmol/mg of bacterial protein.

### RNA extraction and northern blotting

*Y. pestis* strains were grown in LB medium at 26 °C to an OD_620_ of about 1.0 and stored at 4 °C for 8 h. The bacterial cultures were diluted 1:20 into 18 mL LB medium for cultivation to middle exponential phase (OD_620_ ≈ 1.0) at 26 °C. Before harvesting, bacterial cultures were mixed with double-volume RNAprotect Regent (Qiagen) to minimize RNA degradation. Total RNA was isolated using TRIzol Reagent (Invitrogen). The quantity and quality of RNA were determined by NANODROP spectrophotometry (Thermo Scientific). Northern blotting was carried out using a DIG Northern Starter Kit (Roche) following the manufacturer’s protocol as described by Beckmann et al. [40]. Total RNA samples (5–10 μg) were denatured at 95 °C for 3 min, separated on 6 % polyacrylamide-7 M urea gels, and RNA samples were transferred onto Hybond N^+^ membranes (GE Healthcare) by electroblotting. The membranes were UV-crosslinked and pre-hybridized for 1 h, and DIG-labeled RNA probes generated using the primers HmsA/HmsB-NB-F/R were added. The membranes were then hybridized overnight at 68 °C in DIG Easy Hyb buffer (Roche) according to the manufacturer’s protocols. Multiple exposures to X-ray film were taken to achieve the desired signal strength. RNA probes were synthesized by in vitro transcription using T7 RNA polymerase, then RNA was immunologically detected and scanned.

### Primer extension assay

Primer extension assays were performed using a primer extension system-AMV reverse transcriptase kit (Promega) as previously reported by our group [41] with slight modifications. Total RNA (10 μg) was reverse-transcribed using the ^32^P-labelled primer HmsA-PE-R. The cDNA products were subjected to electrophoresis in a 6 % polyacrylamide-8 M urea gel. The gel was then analyzed by autoradiography (Kodak film). To serve as sequence ladders, sequencing reactions were also performed with the same primers used for primer extension, using the AccuPower & Top DNA Sequencing Kit (Bioneer).

### β-galactosidase assay

Genes with a promoter-proximal DNA region were cloned into the low-copy-number transcriptional fusion vector pRW50 [42], which harbors a promoterless *lacZ* reporter gene. The recombinant plasmid or the empty pRW50 (negative control) was transformed into *Y. pestis* strains and the β-Galactosidase Enzyme Assay System (Promega) was used to measure β-galactosidase activity in three independent cellular extracts [18].

### Quantitative RT-PCR (qRT-PCR)

The cDNA was synthesized from 5 μg of RNA using the ThermoScrip RT-PCR System (Invitrogen). Real-time PCR was performed in duplicate for each RNA sample using the TransStart™ Green qPCR SuperMix UDG (TransGen Biotech) with an appropriate cDNA dilution as a template. Three biological replicates were performed for each strain. Control reactions were carried out in parallel in the absence of the reverse transcriptase, 16S rRNA was used as an internal standard to normalize the expression levels of the tested sRNA candidates. Relative quantitative analysis was performed across different cDNA templates using the LightCycler 480 software (Bio-Rad).

## Results

### Enhanced role of HmsA in biofilm formation

**Fig. 1:**
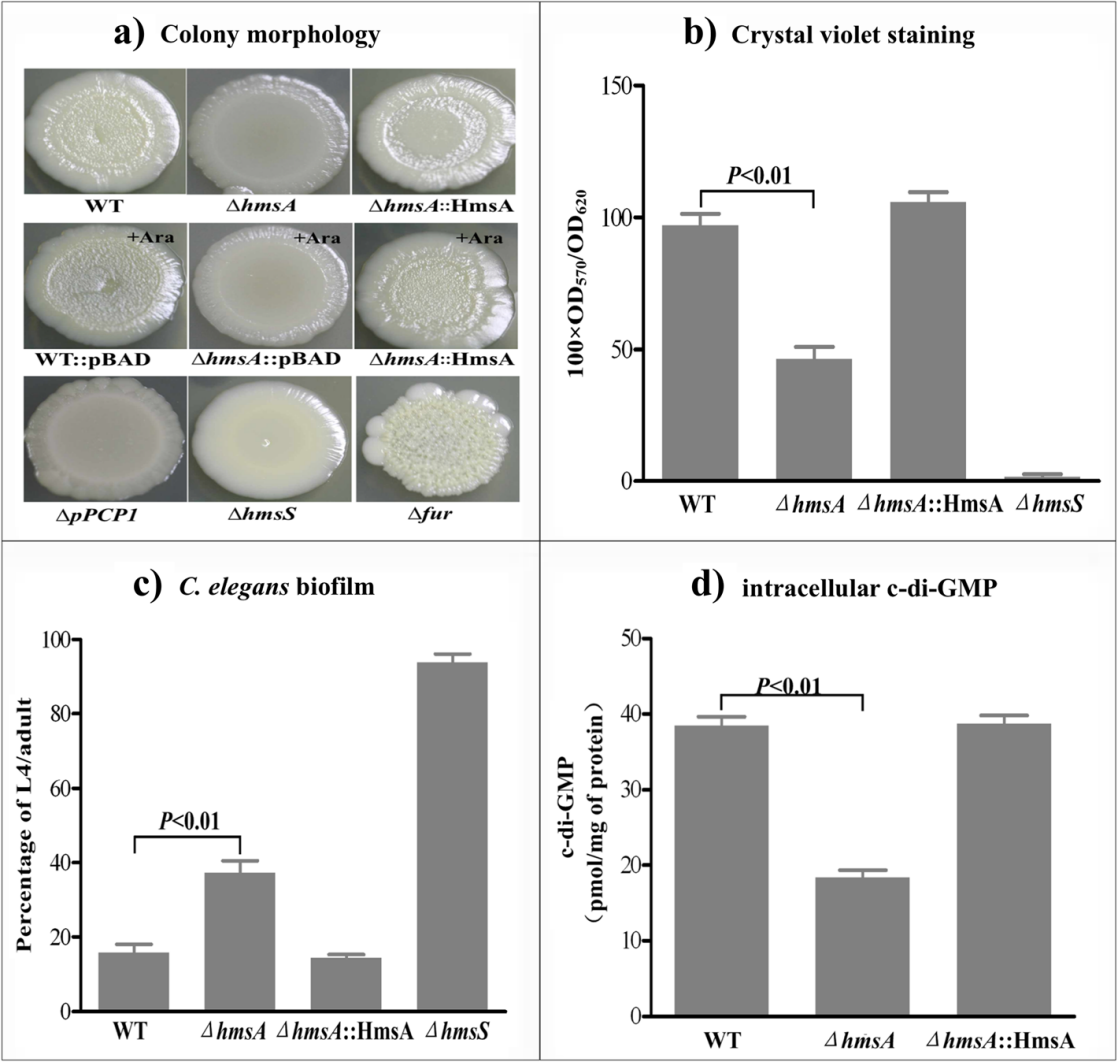
Biofilm formation capacity and c-di-GMP production mediated by HmsA. **a** Bacterial colony morphology. The WT::pBAD, ∆*hmsA*::pBAD and ∆*hmsA*::HmsA strains were incubated on LB agar with 0.1 % arabinose designated as “+Ara” (*middle row*). The ∆*hmsS* or ∆*fur* strains were used as negative or positive controls for this assay, respectively (*bottom row*). **b** Crystal violet staining. Biofilms were quantified by crystal violet staining in the WT, ∆*hmsA* and ∆*hmsA*::HmsA strains grown in LB medium with the addition of 0.1 % arabinose. The ∆*hmsS* strain was grown under the same conditions as the negative control. Data are presented as the average of three separate experiments and error bars represent the standard deviation. **c** *C. elegans* biofilms. The percentage of L4/adults after incubation of nematode eggs on a lawn of the indicated *Y. pestis* strains was used to evaluate the capacity for biofilm formation. **d** Intracellular c-di-GMP concentration

HmsA, a pPCP1 plasmid-derived sRNA, was previously identified as an Hfq-dependent sRNA in *Y. pestis* by RNA-seq in our laboratory. To determine the function of HmsA, we constructed a *hmsA* deletion mutant Δ*hmsA* and an inducible HmsA-overexpressed strain Δ*hmsA*::HmsA. The pBAD promoter driving *hmsA* expression from pBAD-TF was induced by the addition of 0.1 % arabinose. We found that deletion of *hmsA* did not affect the growth rate of *Y. pestis* strain 201 in LB medium (Additional file 1: Figure S1). Colony morphology was much smoother in pPCP1-null *Y. pestis* than WT strain 201 (Fig. 1a), suggesting the role of plasmid pPCP1 in the biofilm phenotype. To further investigate the contribution of HmsA to the regulation of biofilm formation in *Y. pestis*, the rugose colony assay was initially performed. The *∆fur* or *∆hmsS* mutants were used as positive and negative controls, respectively, for the biofilm formation assays. The relatively smooth colonies of *Y. pestis* Δ*hmsA* were compared with those of the WT strain (Fig. 1a). Smooth colony morphology was exhibited by the HmsA overexpressed strain upon induction with arabinose, suggesting that HmsA positively regulates expression of biofilm-associated genes.

The role of HmsA as a regulator of biofilm formation was subsequently confirmed by crystal violet staining and nematode development assays. Quantitative determination by crystal violet staining revealed an approximately 50 % decrease in bacterial biofilm of the *Y. pestis* Δ*hmsA* mutant at the air-liquid interface compared with the WT strain and the Δ*hmsA*::HmsA strain (Fig. 1b). Incubation of nematode eggs on bacterial lawns of the WT and Δ*hmsA*::HmsA strain, revealed that only a small percentage (below 20 %) of eggs grew to L4/adult nematodes due to abundant attachment of biofilms to nematode heads. By contrast, bacterial lawns of the ∆*hmsA* and ∆*hmsS* mutant strains allowed the growth of about 40 % and 95 % of eggs into L4/adult nematodes, respectively (Fig. 1c). These results indicated that HmsA enhances biofilm formation in *Y. pestis*.

### Impact of HmsA on the biosynthesis of c-di-GMP molecules

Biofilm formation in *Y. pestis* is regulated by the intracellular concentration of the second messenger c-di-GMP. As HmsA influences the extent of biofilm formation by *Y. pestis*, we hypothesized that deletion of *hmsA* would result in decreased c-di-GMP levels. To test the effect of HmsA on c-di-GMP synthesis in *Y. pestis* cells, the concentration of c-di-GMP was determined in the WT, ∆*hmsA* and Δ*hmsA*::HmsA strains by HPLC-MS/MS. The production of c-di-GMP was clearly reduced in the ∆*hmsA* mutant compared with the WT and Δ*hmsA*::HmsA mutant (Fig. 1d). The c-di-GMP levels were comparable between the WT and Δ*hmsA*::HmsA strain grown under the same conditions. However, absence of the *hmsA* gene resulted in a reduction in c-di-GMP levels by about 50 %. These data indicated that HmsA plays a role in modulation of c-di-GMP levels, and that c-di-GMP levels correlate with the amount of biofilm produced.

### HmsA affects the transcriptional regulation of structural genes for EPS synthesis

Most sRNAs derived from intergenic regions act as *trans*-acting sRNAs repressing translation and destroying mRNA stability via base pairing with their target mRNAs. Translational repression of a target mRNA results in active or passive mRNA degradation. We used a primer extension assay and RT-PCR to determine the HmsA-mediated regulatory effects on biofilm-related genes at the transcriptional and post-transcriptional level in *Y. pestis*. A *lacZ* transcriptional fusion assay was also performed to explore the possibility that abundance changes occur at the transcriptional level.

**Fig. 2:**
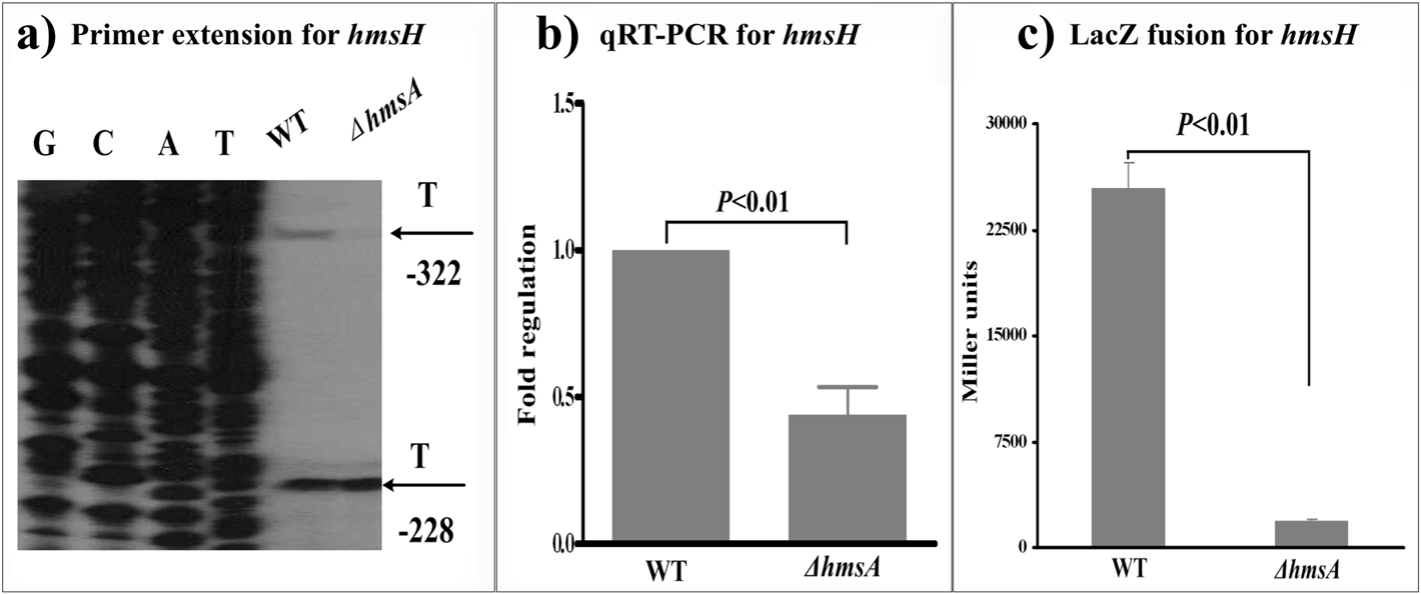
Regulatory effects of HmsA on *hmsH.* **a** Primer extension results. The relative levels of *hmsH* transcript were determined in the WT and ∆*hmsA* mutant strains by primer extension assays. The Sanger sequence ladders (lanes G, C, A and T) and the primer extension products of *hmsH* were analyzed on an 8 M urea-6 % acrylamide sequencing gel. The transcription start sites of *hmsH* were indicated by arrows, and the minus number under the arrow indicates the nucleotide position upstream of the *hmsH* start codon. **b** qRT-PCR results. The relative levels of the *hmsH* transcript were determined in the WT and ∆*hmsA* mutant strains by qRT-PCR. **c** *lacZ* fusion results. The *hmsH*::*lacZ* transcriptional fusion vector was transformed into the WT and ∆*hmsA* mutant strains. The transcriptional activity of *hmsH* determined in the bacterial cellular extracts was represented as Miller units of β-galactosidase activity

The main component of *Y. pestis* biofilm, EPS, is encoded by the structural *hmsHFRS* operon. To investigate whether HmsA affects the mRNA level of *hmsHFRS*, the first gene *hmsH* was chosen for further analysis of transcriptional regulation in the WT and Δ*hmsA* mutant strain (Fig. 2). Relative to the WT strain, the absence of *hmsA* resulted in a decrease in *hmsH* transcript. However, the *lacZ* fusion assay indicated that *hmsH* is significantly downregulated upon deletion of HmsA. Therefore, the regulatory effects of HmsA on the *hmsH* gene appear to operate at the transcriptional level rather than the post-transcriptional level.

### HmsA affects the regulation of genes for c-di-GMP synthesis and degradation

**Fig. 3:**
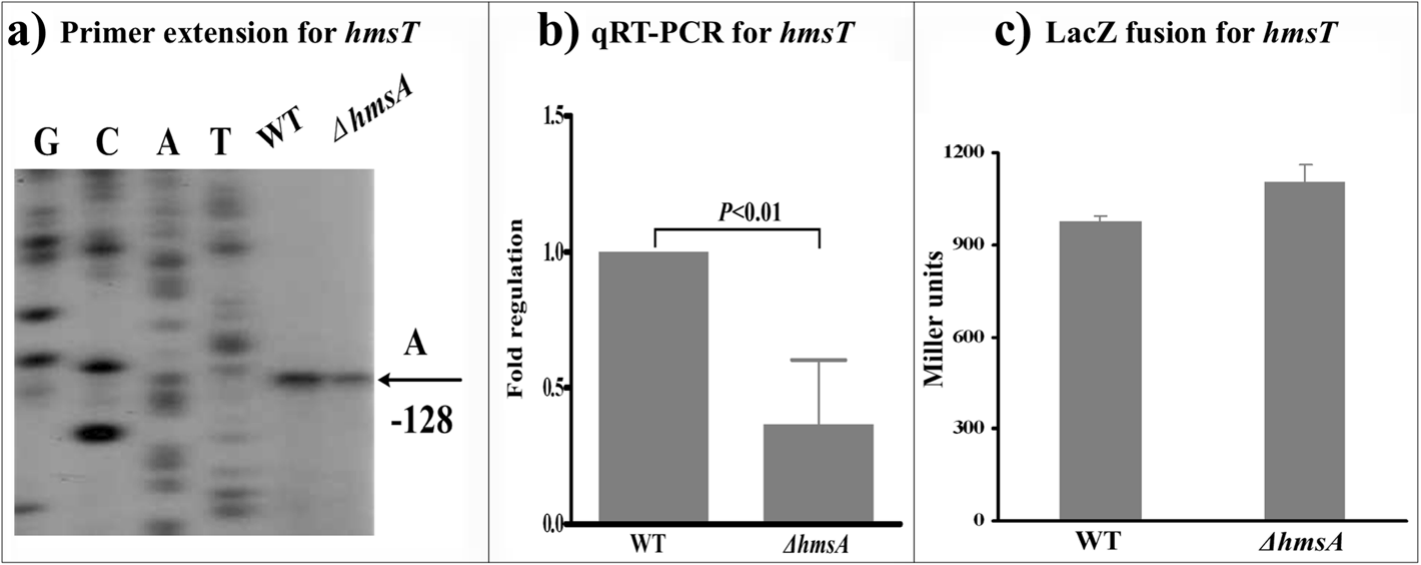
Regulatory effects of HmsA on *hmsT*. **a** Primer extension results. The relative levels of *hmsH* transcript were determined in the WT and ∆*hmsA* mutant strains by primer extension assays. The Sanger sequence ladders (lanes G, C, A and T) and the primer extension products of *hmsH* were analyzed on an 8 M urea-6 % acrylamide sequencing gel. The transcription start sites of *hmsH* were indicated by arrows, and the minus number under the arrow indicates the nucleotide position upstream of the *hmsH* start codon. **b** qRT-PCR results. The relative levels of the *hmsH* transcript were determined in the WT and ∆*hmsA* mutant strains by qRT-PCR. **c** *lacZ* fusion results. The *hmsH*::*lacZ* transcriptional fusion vector was transformed into the WT and ∆*hmsA* mutant strains. The transcriptional activity of *hmsH* determined in the bacterial cellular extracts was represented as Miller units of β-galactosidase activity

**Fig. 4:**
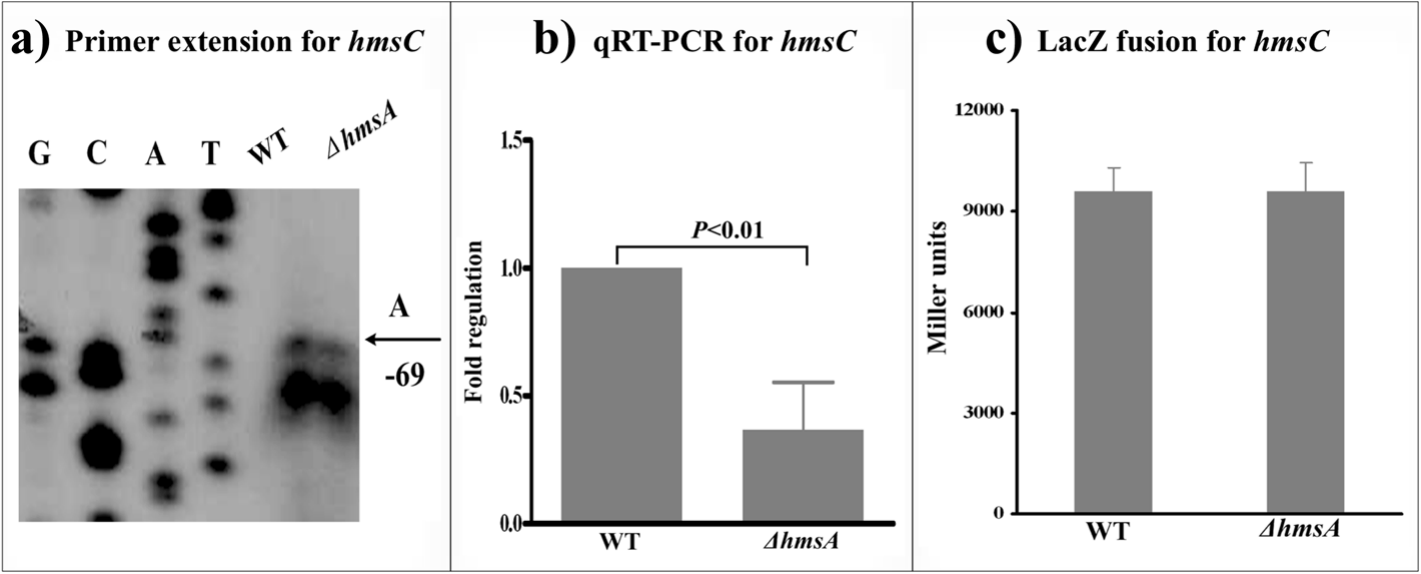
Regulatory effects of HmsA on *hmsC*. **a** Primer extension results. The relative levels of *hmsH* transcript were determined in the WT and ∆*hmsA* mutant strains by primer extension assays. The Sanger sequence ladders (lanes G, C, A and T) and the primer extension products of *hmsH* were analyzed on an 8 M urea-6 % acrylamide sequencing gel. The transcription start sites of *hmsH* were indicated by arrows, and the minus number under the arrow indicates the nucleotide position upstream of the *hmsH* start codon. **b** qRT-PCR results. The relative levels of the *hmsH* transcript were determined in the WT and ∆*hmsA* mutant strains by qRT-PCR. **c** *lacZ* fusion results. The *hmsH*::*lacZ* transcriptional fusion vector was transformed into the WT and ∆*hmsA* mutant strains. The transcriptional activity of *hmsH* determined in the bacterial cellular extracts was represented as Miller units of β-galactosidase activity

**Fig. 5:**
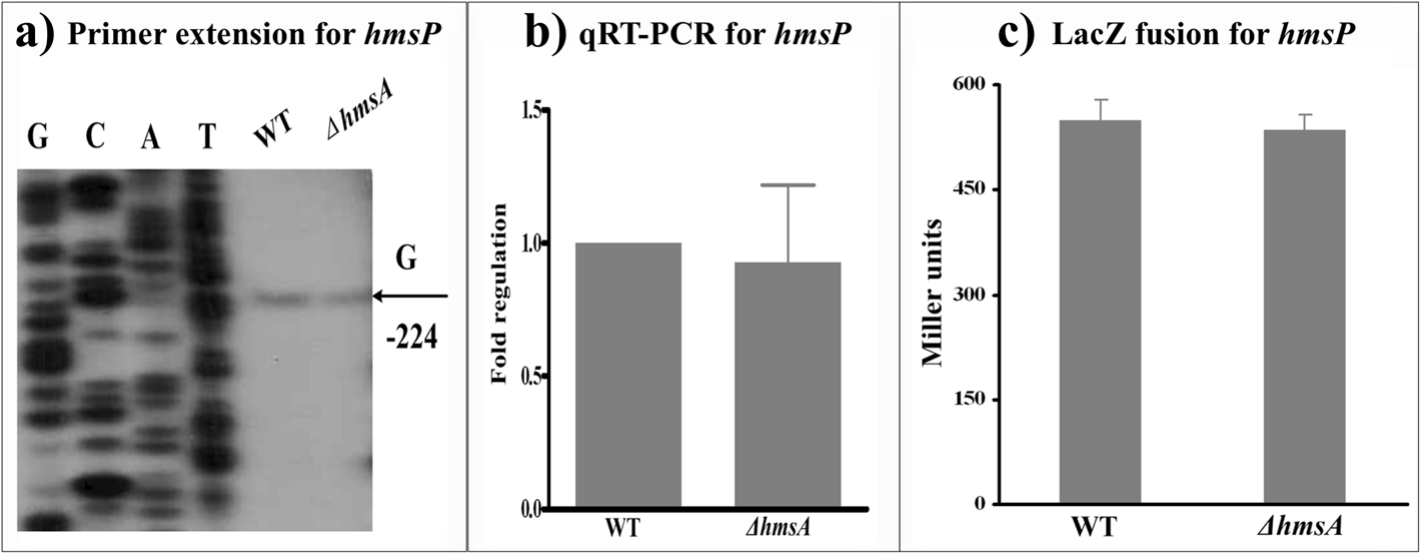
Regulatory effects of HmsA on *hmsP*. **a** Primer extension results. The relative levels of *hmsH* transcript were determined in the WT and ∆*hmsA* mutant strains by primer extension assays. The Sanger sequence ladders (lanes G, C, A and T) and the primer extension products of *hmsH* were analyzed on an 8 M urea-6 % acrylamide sequencing gel. The transcription start sites of *hmsH* were indicated by arrows, and the minus number under the arrow indicates the nucleotide position upstream of the *hmsH* start codon. **b** qRT-PCR results. The relative levels of the *hmsH* transcript were determined in the WT and ∆*hmsA* mutant strains by qRT-PCR. **c** *lacZ* fusion results. The *hmsH*::*lacZ* transcriptional fusion vector was transformed into the WT and ∆*hmsA* mutant strains. The transcriptional activity of *hmsH* determined in the bacterial cellular extracts was represented as Miller units of β-galactosidase activity

*Y. pestis* contains two genes (*hmsT* and *hmsD*) encoding diguanylate cyclase enzymes and one gene (*hmsP*) encoding the phosphodiesterase enzyme, which all play a role in determining the intracellular level of c-di-GMP. To investigate the regulatory role of HmsA on the *hmsT* and *hmsD* genes, the expression levels of *hmsT* and *hmsC* (the first gene of the *hmsCDE* operon) were measured as described for *hmsH* (Figs. 3 and 4). The abundance of both *hmsT* and *hmsC* transcripts was reduced in the Δ*hmsA* strain compared with the WT strain. However, we found no significant differences in transcriptional activity between the WT and Δ*hmsA* strains, suggesting that both *hmsT* and *hmsD* are likely activated by HmsA at the post-transcriptional level. By contrast, deletion of HmsA did not significantly alter the levels of *hmsP* transcript (Fig. 5) indicating that the abundance of the *hmsP* transcript is correlated with HmsA.

### HmsA affects the regulation of the biofilm-associated sRNA HmsB

**Fig. 6:**
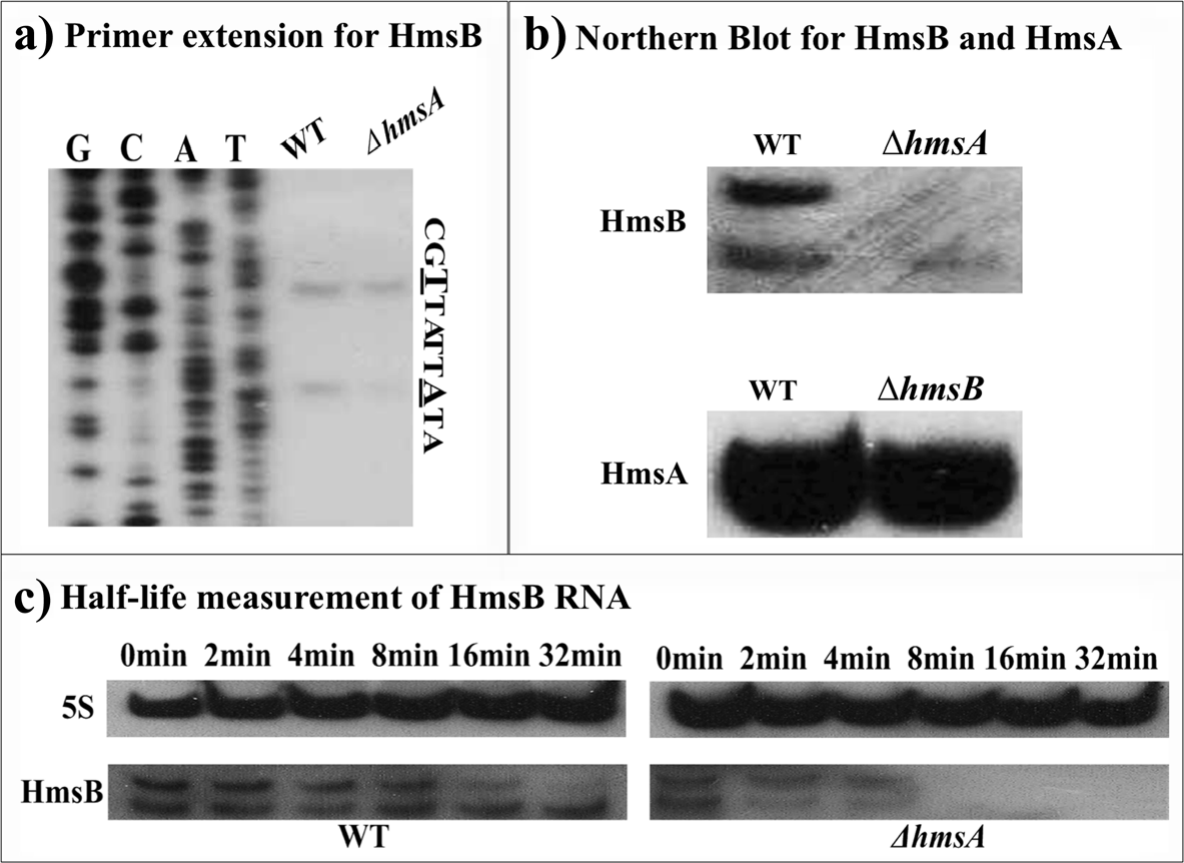
Regulatory effects of HmsA on HmsB. **a** Primer extension results. **b** Confirmation of HmsA and HmsB by northern blot analysis. The transcript of *hmsB/hmsA* in the WT and ∆*hmsA/*∆*hmsB* mutant strains was monitored by northern blotting (*upper and bottom panel*). **c** Measurement of the half-life of HmsB. The WT and ∆*hmsA* strains were grown to exponential phase at 26 °C and then treated with 250 μg/mL of rifampicin. Culture samples were collected at 0, 2, 4, 8, 16 and 32 min and were subject to RNA extraction and northern blotting using 5S rRNA and HmsB probes

The sRNA HmsB has been recently identified as a biofilm activator by increasing EPS production in *Y. pestis* [35]. Using the same RNA extracted for the primer extension assay, the mRNA abundance of HmsB and HmsA was investigated by northern blot analysis in the WT and Δ*hmsA* or Δ*hmsB* mutant strains (Fig. 6a and b). Surprisingly, the level of HmsB transcript sharply decreased in the absence of *hmsA*. However, the level of HmsA transcript remained unchanged in the WT and Δ*hmsB* strains. These data suggested that the transcription of HmsB might be HmsA-dependent but that HmsA transcription is not influenced by HmsB. To further clarify whether HmsA alters the stability of HmsB, the half-life of the HmsB transcript was measured in the WT and Δ*hmsA* mutant strains by northern blot analysis (Fig. 6c). HmsB was relatively stable for 30 min in the WT strain, but was rapidly degraded in the Δ*hmsA* mutant, suggesting that HmsA might affect the stability of HmsB in *Y. pestis.*

### Regulatory effects of HmsA on biofilm-related regulators (RovA and RovM) and the ferric uptake regulator (Fur)

**Fig. 7:**
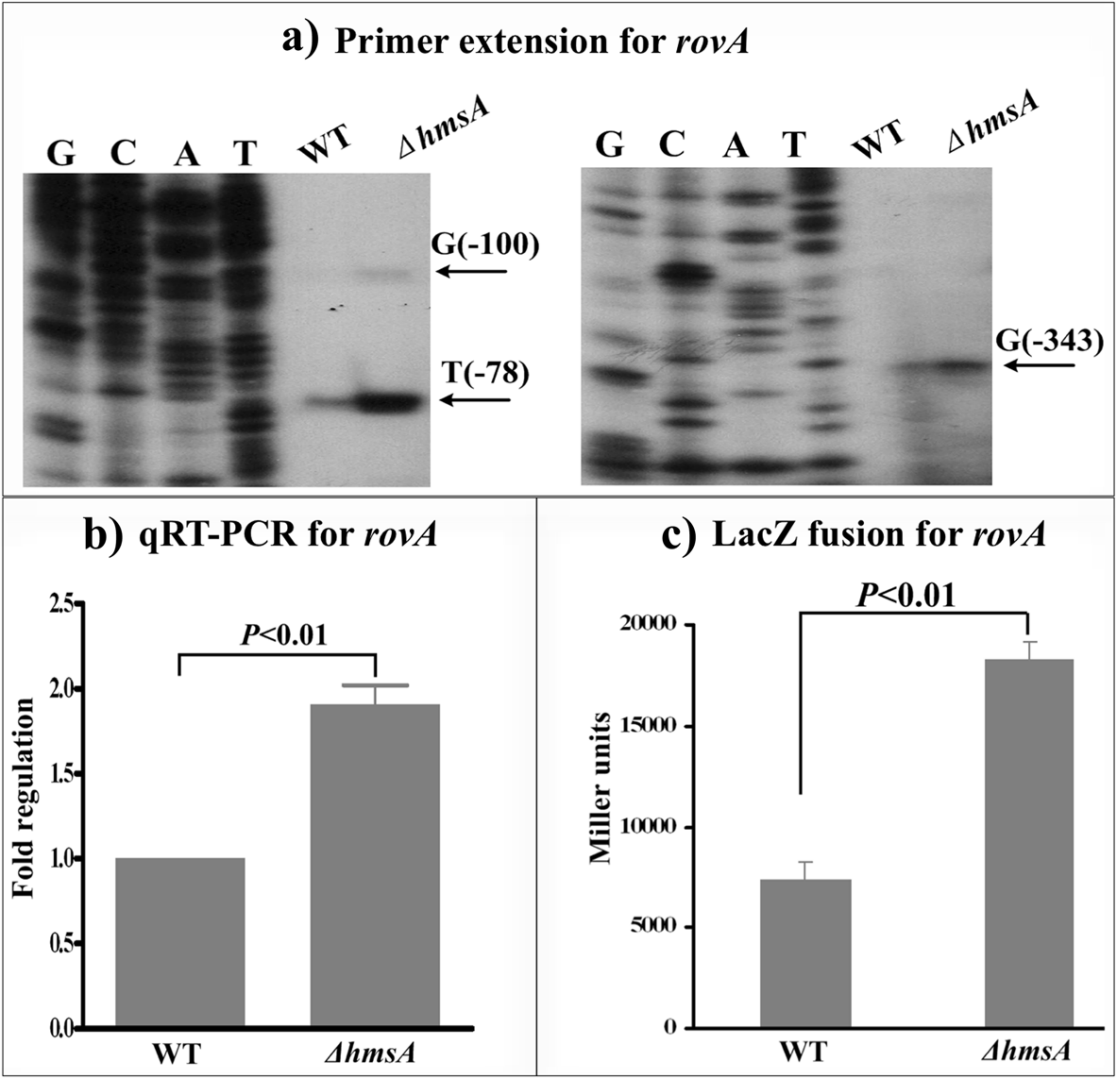
Regulatory effects of HmsA on *rovA*. **a** Primer extension results. The relative levels of *hmsH* transcript were determined in the WT and ∆*hmsA* mutant strains by primer extension assays. The Sanger sequence ladders (lanes G, C, A and T) and the primer extension products of *hmsH* were analyzed on an 8 M urea-6 % acrylamide sequencing gel. The transcription start sites of *hmsH* were indicated by arrows, and the minus number under the arrow indicates the nucleotide position upstream of the *hmsH* start codon. **b** qRT-PCR results. The relative levels of the *hmsH* transcript were determined in the WT and ∆*hmsA* mutant strains by qRT-PCR. **c** *lacZ* fusion results. The *hmsH*::*lacZ* transcriptional fusion vector was transformed into the WT and ∆*hmsA* mutant strains. The transcriptional activity of *hmsH* determined in the bacterial cellular extracts was represented as Miller units of β-galactosidase activity

**Fig. 8:**
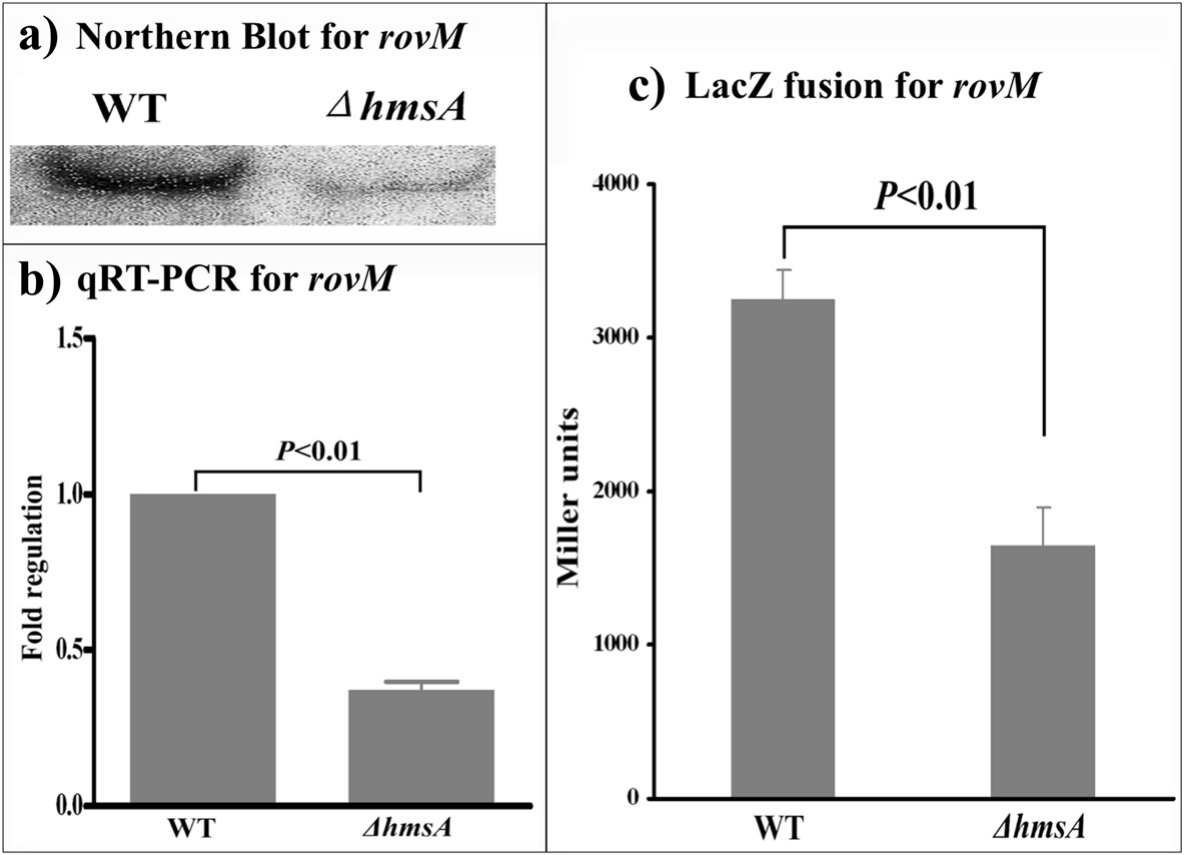
Regulatory effects of HmsA on *rovM*. **a** Primer extension results. The relative levels of *hmsH* transcript were determined in the WT and ∆*hmsA* mutant strains by primer extension assays. The Sanger sequence ladders (lanes G, C, A and T) and the primer extension products of *hmsH* were analyzed on an 8 M urea-6 % acrylamide sequencing gel. The transcription start sites of *hmsH* were indicated by arrows, and the minus number under the arrow indicates the nucleotide position upstream of the *hmsH* start codon. **b** qRT-PCR results. The relative levels of the *hmsH* transcript were determined in the WT and ∆*hmsA* mutant strains by qRT-PCR. **c** *lacZ* fusion results. The *hmsH*::*lacZ* transcriptional fusion vector was transformed into the WT and ∆*hmsA* mutant strains. The transcriptional activity of *hmsH* determined in the bacterial cellular extracts was represented as Miller units of β-galactosidase activity

**Fig. 9:**
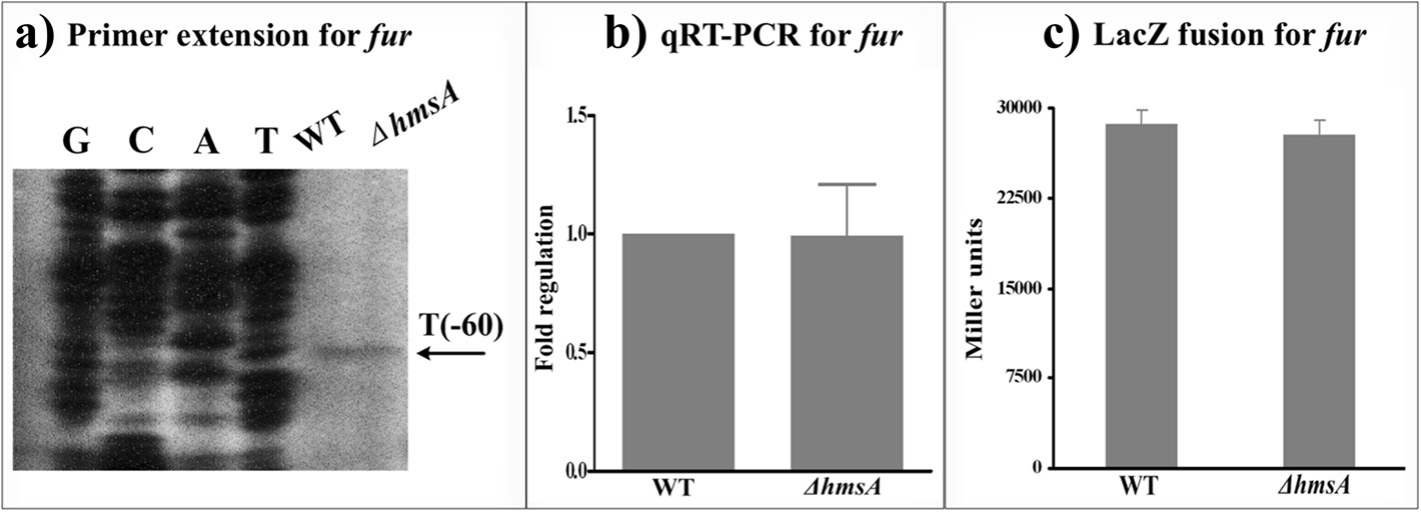
Regulatory effects of HmsA on *fur*. **a** Primer extension results. The relative levels of *hmsH* transcript were determined in the WT and ∆*hmsA* mutant strains by primer extension assays. The Sanger sequence ladders (lanes G, C, A and T) and the primer extension products of *hmsH* were analyzed on an 8 M urea-6 % acrylamide sequencing gel. The transcription start sites of *hmsH* were indicated by arrows, and the minus number under the arrow indicates the nucleotide position upstream of the *hmsH* start codon. **b** qRT-PCR results. The relative levels of the *hmsH* transcript were determined in the WT and ∆*hmsA* mutant strains by qRT-PCR. **c** *lacZ* fusion results. The *hmsH*::*lacZ* transcriptional fusion vector was transformed into the WT and ∆*hmsA* mutant strains. The transcriptional activity of *hmsH* determined in the bacterial cellular extracts was represented as Miller units of β-galactosidase activity

RovM has been defined as a virulence regulator and represses the activity of the RovA regulator, which is also able to regulate biofilm formation in *Y. pseudotuberculosis* and *Y. pestis* [43–45]. RovA has been shown to repress biofilm formation by negatively regulating *hmsT* in *Y. pestis* [21]. The ferric uptake regulator (Fur) is a global regulator of iron homeostasis that contributes to biofilm formation in bacteria [46]. In this study, we monitored changes in the abundance and transcriptional activity of *rovA*, *rovM* and *fur* transcripts in the WT and Δ*hmsA* mutant strains. Our results showed that the mRNA level of *rovA* in the ∆*hmsA* mutant strain was significantly increased compared with that of the WT strain (Fig. 7). By contrast, the mRNA level of *rovM* in the ∆*hmsA* strain was significantly decreased, as shown by both northern blotting and qRT-PCR. We also observed the reduced transcriptional activity of *rovM* in the ∆*hmsA* strain relative to the WT strain (Fig. 8). This suggested that HmsA carries out transcriptional regulation of *rovA* and *rovM* via different mechanisms. No change in the abundance of the *fur* transcript was detected in the WT and Δ*hmsA* strains (Fig. 9). Taken together, these data are all consistent with a HmsA-mediated biofilm phenotype.

## Discussion

*Y. pestis* evolved from *Y. pseudotuberculosis* by acquiring novel pathogenic traits, which conferred the ability to cause fatal disease in mammals, and the capacity to form a biofilm in fleas [8, 41, 47]. Two laterally acquired plasmids, pPCP1 and pMT1, have been shown to be essential for *Y. pestis* pathogenesis and flea transmission, respectively [6–8]. Here we present evidence that plasmid pPCP1 contributes to biofilm formation in *Y. pestis*. Our study showed that HmsA was implicated in chromosome-encoded regulatory networks of biofilm formation by modulating the concentration of c-di-GMP and EPS production. Until now, two regulatory sRNAs were reported to affect biofilm formation in *Y. pestis*, which constituted a new class of regulators of biofilm matrix production. Strikingly, the stability of HmsB appeared to be impaired by HmsA. A consecutive 13-nt base pairing region was found at the 5ʹ-terminal region of HmsA and within HmsB using the IntaRNA program (Additional file 2: Figure S2a). Whether the potentially direct interaction between HmsA and HmsB accounts for blocking of the accessibility to ribonuclease remains to be experimentally determined.

**Fig. 10:**
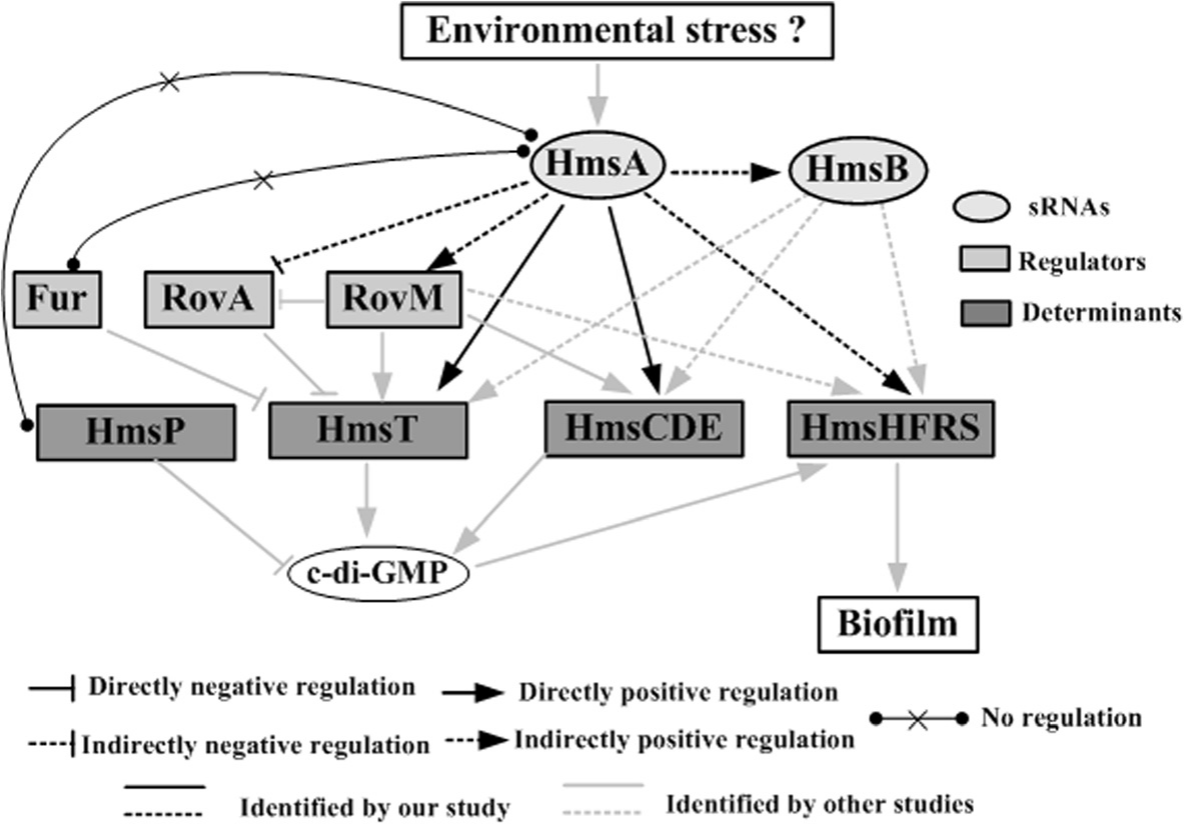
Regulatory networks of HmsA on biofilm formation

*Trans*-acting sRNAs represent a major class of sRNAs in bacteria. The RNA-binding protein Hfq usually aids the sRNA-mRNA interaction and the stability of sRNAs [48, 49]. HmsA was confirmed as a Hfq-dependent sRNA in our previous study [28]. This promoted us to hypothesize that HmsA would function by interacting with target mRNAs at the post-transcriptional level. To clarify the genetic mechanism by which HmsA promotes biofilm formation, dozens of biofilm-associated genes were chosen for further analysis of HmsA-mediated regulation. We investigated the potential regulatory role of HmsA on related genes at the transcriptional and post-transcriptional level. Our results showed that the loss of *hmsA* leads to changes in mRNA abundance of a novel sRNA (HmsB), several canonical biofilm-enhancing factor genes (*hmsHFRS*, *hmsT* and *hmsCDE*) and transcriptional regulator genes (*rovM* and *rovA*). Subsequently we showed that biofilm phenotype mediated by HmsA is likely due to the transcriptional regulation of HmsB, HmsHFRS, RovA and RovM and post-transcriptional activation of HmsT and HmsD. In this study we focused on the mechanism by which HmsA influenced biofilm formation in *Y. pestis*, and the regulatory networks are summarized in Fig. 10.

Notably, most of the tested genes were transcriptionally regulated, suggesting the indirect role of HmsA. Only *hmsT* and *hmsCDE* appeared to be post-transcriptionally regulated. In general, sRNAs promote translational activation by either increasing accessibility of the ribosomal binding site on mRNA or adjusting mRNA susceptibility to ribonucleolytic degradation [50]. We predicted the secondary structure of the 5' UTR of HmsT/HmsC and potential interactions between HmsA and HmsT/HmsC using the Mfold (http://mfold.rit.albany.edu) algorithm and the IntaRNA program (http://rna.informatik.uni-freiburg.de/IntaRNA), respectively. The stem-loop structure was found to be located close to the ribosomal binding site or translational start site of both HmsT and HmsC (Additional file 2: Figure S2b and S2c). Interestingly, the interactive sites between HmsA and HmsT/HmsC partially overlap with the stem-loop-forming sites of HmsT/HmsC, which is consistent with the traditional view of sRNA-mediated target activation. However, the precise mechanism by which the *hmsT* and *hmsD* transcripts, as well as other mRNAs, are directly targeted by HmsA remains to be further investigated.

## Conclusion

In this study the plasmid pPCP1-deriving sRNA HmsA was characterized as an activator of biofilm formation in *Y. pestis*. The biofilm-associated genes were found regulated by HmsA, implying that sRNAs encoded on the laterally acquired plasmids might involve in the chromosome-based regulatory networks. It provides a further insight into importance of sRNAs in Y. pestis-specific physiology and evolution.

## Additional files

Additional file 1: Figure S1. Characterization of sRNA HmsA in *Y. pestis.* a) Promoter analysis and HmsA expression determined by RNA-seq data. b) Primer extension analysis of HmsA in *Y. pestis* WT and ∆*hfq* mutant strains. c) Growth curves of the WT and Pdr1 mutant strains. (TIFF 833 kb) Additional file 2: Figure S2. Predicted interactions of HmsA with HmsB, *hmsC* and *hmsT.* The interaction between HmsA and HmsB predicted by IntaRNA is shown in a). Predicted structures of the 5′ UTR of *hmsC* and *hmsT* mRNA are shown in b) and c), respectively. The start codon is boxed and the ribosomal binding site is underlined. The numbers in the figure indicate the position relative to the start codon (+1) of mRNA. Regions that potentially base pair with HmsA are underlined in blue. (TIFF 1118 kb)
