# Supplementary figures and images for: Plasmid pPCP1-derived sRNA HmsA promotes biofilm formation of Yersinia pestis
Source: BMC Microbiol. 2016 Aug 4;16:176. doi: 10.1186/s12866-016-0793-5 (PMC4973556; doi:10.1186/s12866-016-0793-5)

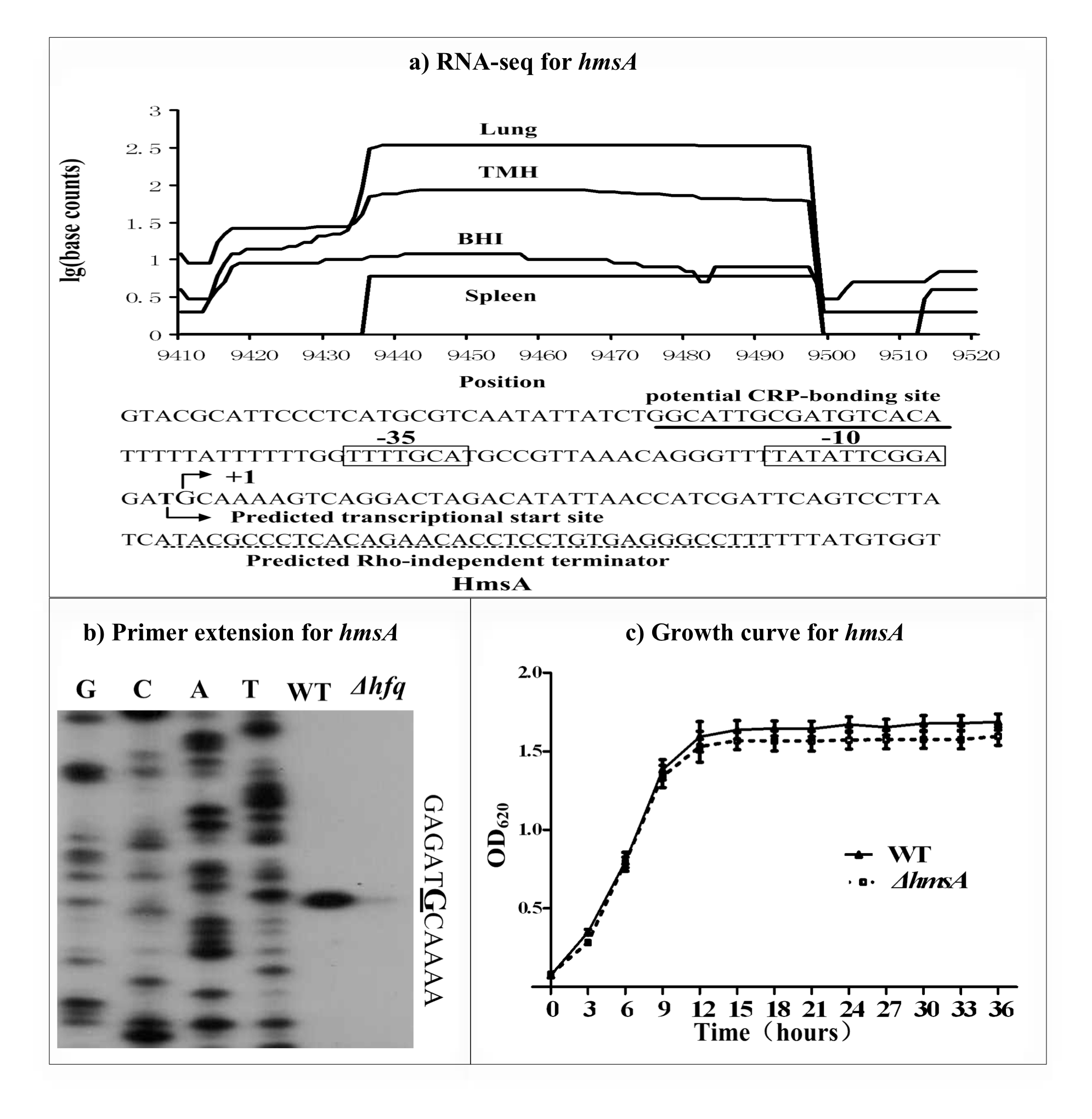

Supplement: Additional file 1: Figure S1. — Characterization of sRNA HmsA in Y. pestis. a) Promoter analysis and HmsA expression determined by RNA-seq data. b) Primer extension analysis of HmsA in Y. pestis WT and ∆hfq mutant strains. c) Growth curves of the WT and Pdr1 mutant strains. (TIFF 833 kb) [file 12866_2016_793_MOESM1_ESM.tiff]

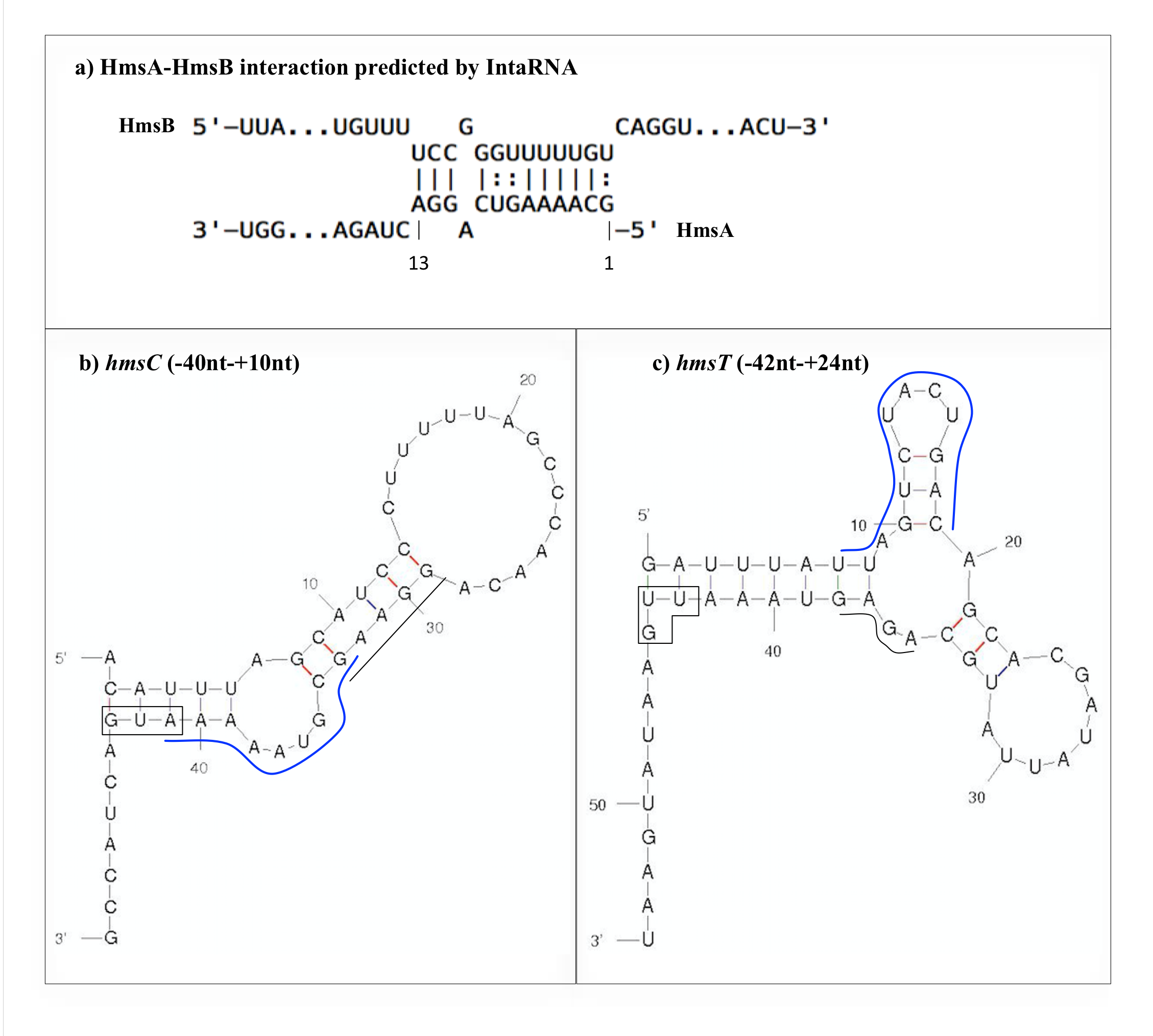

Supplement: Additional file 2: Figure S2. — Predicted interactions of HmsA with HmsB, hmsC and hmsT. The interaction between HmsA and HmsB predicted by IntaRNA is shown in a). Predicted structures of the 5′ UTR of hmsC and hmsT mRNA are shown in b) and c), respectively. The start codon is boxed and the ribosomal binding site is underlined. The numbers in the figure indicate the position relative to the start codon (+1) of mRNA. Regions that potentially base pair with HmsA are underlined in blue. (TIFF 1118 kb) [file 12866_2016_793_MOESM2_ESM.tiff]
